# Dentistry during the COVID-19 Epidemic: An Italian Workflow for the Management of Dental Practice

**DOI:** 10.3390/ijerph17093325

**Published:** 2020-05-11

**Authors:** Matteo Peditto, Simone Scapellato, Antonia Marcianò, Paola Costa, Giacomo Oteri

**Affiliations:** 1 Postgraduate School of Oral Surgery, Department of Biomedical, Dental Sciences and Morphofunctional Imaging, University of Messina, 98125 Messina, Italy; mpeditto@unime.it (M.P.); simonescapellato@hotmail.com (S.S.); pa.costa@hotmail.it (P.C.); 2 Department of Clinical and Experimental Medicine, University of Messina, 98125 Messina, Italy; antmarciano@unime.it

**Keywords:** dentistry, COVID-19, epidemic, infection control, prevention, dental management, workflow, dental procedure, preventive measures

## Abstract

The COVID-19 outbreak has raised concerns about infection control all over the world. Among health workers, dentists are particularly exposed to the COVID-19 infection risk. The aim of this paper is to present a workflow to manage dental procedures already in use at the Dental Unit of the University Hospital of Messina. The proposed workflow accounts for the many aspects of dental practitioners’ risk in the COVID-19 era, and focuses on the assessment of patient risk level, a two-phase dental procedure management (remote and face-to-face), and the use of specific preventive measures. No cases of COVID-19 infection were detected among patients and staff of the dental unit in a two-month period of time while using this protocol. This workflow seems a promising and effective solution to manage dental procedures during the COVID-19 outbreak, and could be implemented in both public and private practices until the emergency is contained.

## 1. Introduction

In December 2019, in Wuhan city (China), a pneumonia of unknown cause was detected and first reported to the WHO Country Office in China on 31 December 2019 [1]. This pneumonia infection has rapidly spread from Wuhan to most other Chinese provinces and other 24 countries [2,3]. On 30 January 2020, the outbreak was declared as a Public Health Emergency of International Concern [1]. Chinese researchers have quickly discovered and isolated a novel coronavirus, (2019-nCoV), responsible for the onset of pneumonia [4]. On 11 February 2020, WHO announced a name for the new coronavirus disease, COVID-19 [1], and increased the assessment of the risk of spread to “very high” on 28 February 2020 [5]. On 11 March 2020, WHO General Manager defined the spread of COVID-19 no longer confined to certain geographical areas, but a pandemic spread all over the world [5]. The first two cases of COVID-19 in Italy, a couple of Chinese tourists, were confirmed on 30 January 2020 by the Spallanzani Institute (Rome) where they were hospitalized in isolation until their recovery on 26 February 2020. The first Italian case of secondary transmission occurred in Codogno in Lombardy on 18 February 2020 [6].

The whole world is fighting against the spread of COVID-19; the WHO Coronavirus disease 2019 (COVID-19) Situation Report–94 reported over 2,500,000 cases and over 175,000 deaths [7]. As of 23 April 2020, 189,973 cases have been reported in Italy (106,848 positives, 57,576 recovered, 25,549 deaths), of which 519 were from Messina [8]. Strict containment measures adopted by governments establish the limitation of people circulating outside their home, social distancing, the cessation of almost all commercial activities, remote working and home schooling routines using videoconferencing platforms (e.g., Microsoft Teams, Skype, Zoom, etc.), and the request to use protective masks and gloves to decrease the risk of infection [9].

### 1.1. COVID-19: Updated Report of the Pandemic

#### 1.1.1. Incubation Period and Symptoms

It has recently been reported in the literature that the incubation period of 2019-nCoV in humans varies from about 2 to 14 days (possible outliers: 0–27 days) [10,11,12]. Viral shedding supposedly begins 2–3 days before symptom onset [13], after which the viral load decreases monotonically. The virus can be detected after 20 days from symptom onset [14], however, the live virus can no longer be cultured after 8 days, suggesting a severe reduction in infectiousness [15,16]. Typical clinical symptoms of the patients who suffered from COVID-19 are fever, cough, and myalgia or fatigue with abnormal chest CT. Less common symptoms are sputum production, headache, hemoptysis and diarrhea [10,17,18].

#### 1.1.2. Transmission Routes

It has been widely documented in the literature that common transmission routes of 2019-nCoV include droplet (particles diameter ≥5 μm) inhalation generated from coughs and sneezes of infected patients, as well as direct contact with oral, nasal and eye mucous membranes [19]. In addition, studies have shown that 2019-nCoV can be transmitted through saliva [20]; a recent study in Hong Kong on a sample of 12 infected patients showed that coronavirus was present in the saliva of 11 subjects (91.7%) [21]. There is no significant evidence regarding airborne transmission (particles diameter <5 μm) of coronaviruses, including SARS-CoV-2, with the exception of aerosol-generating procedures. Two recent studies where air sampling was performed next to a patient with a high COVID-19 viral load in their nasopharyingeal and throat swabs assessed that SARS-CoV-2 cannot be detected in the air at a distance of 10 cm or more from the patient’s chin with or without wearing a surgical mask, even considering environmental contamination [22,23]. Another study on long distance flights demonstrated that there is no current evidence regarding airborne transmission, supporting the droplet mechanisms instead as the main route of the COVID-19 spread [24]. However, a similar study by researchers in Nebraska found viral RNA in nearly two-thirds of air samples collected in isolation rooms in a hospital treating people with severe COVID-19 and in a quarantine facility housing those with mild infections. While none of the air samples were infectious in cell culture, data suggest that viral aerosol particles are produced by individuals that have the COVID-19 disease—even in the absence of cough—thus, it is not possible to completely rule out airborne transmission yet.

Authors from Sichuan University [25] suggested that salivary glands might represent a reservoir for COVID-19 asymptomatic infection. In fact, the expression of Angiotensin-converting enzyme 2 (ACE2), a key receptor for COVID-19, is higher in minor salivary glands than in the lungs. This could explain the occasional lack of symptoms in infected subjects. Moreover, the positive rate of COVID-19 in patients’ saliva can reach up to 100%, and saliva samples can also cultivate the live virus [21,26], thus the potential infectivity of saliva should be strongly considered.

#### 1.1.3. Stability of the Virus

A recent in vitro study evaluated the persistence of 2019-nCoV after its nebulization on surfaces such as plastic or stainless steel. It has been shown the virus remains stable up to 72 h on plastic surfaces and up to 48 h on stainless steel surfaces, although the viral count is significantly reduced and it has not been found if these amounts are sufficient to cause infection [27]. Dry environments likely impair virus stability the most, and alcohol-based disinfectants (e.g., ethanol) significantly reduce infectivity of enveloped viruses like COVID-19 [28].

#### 1.1.4. COVID-19 Management

To date, clinical management of COVID-19 is mainly symptomatic treatment. Severe cases require respiratory assistance with organ support in intensive care. No specific antiviral treatment exists, but antiviral, antimalarial and biological drugs are administered in clinical trials [29].

### 1.2. Covid-19 and Dentistry

#### 1.2.1. Operator’s Risk in Dentistry

OSHA (Occupational Safety and Health Administration) published a note on the worker exposure risk to COVID-19 [30], identifying four risk levels, from low to very high. Very high-risk exposure level includes occupations with a high potential for exposure to known or suspected sources of COVID-19 during specific medical, postmortem, or laboratory procedures. Healthcare and morgue workers performing aerosol-generating procedures fall into this category. Among health care workers, intensive care unit staff, otolaryngologist, and nurses are considered the most exposed to the risk of infection. To date, 116 doctors have died in Italy due to COVID-19 and, among them, 12 dentists. Dental practitioners risk should not be underestimated. Dentists routinely perform several aerosol-generating procedures due to the use of different tools, like dental high-speed turbine, spray handpiece, or piezoelectric scaler. These instruments largely increase the aerosol produced inside the work environment, thus exposing both clinicians and patient to the risk of infection. It is interesting to note that on 15 March 2020, *The New York Times* published a suggestive article describing that dentists are the most exposed workers to the risk of being affected by COVID-19 [31]. During dental procedures, inhalation of aerosols produced by instruments on patients with COVID-19 can determine a high infection risk [32], considering virus transmission routes. Viral particles can be detected in saliva during COVID-19 infection, thus some authors proposed saliva sampling as a reliable diagnostic tool [26]. On behalf of these considerations, all dentistry operators must always be diligent in protecting against the spread of infectious disease [32], as well as note the importance of providing clear and easy guidelines to manage patients and make dental practice safe from any risk [33].

#### 1.2.2. COVID-19 Prevention in Dentistry

It is crucial to improve effective strategies for prevention, especially for dentists, to reduce risk of contagion from COVID-19 [32]. As of today, one of the main challenges in the dental healthcare is the difficulty in the infected patient identification, due to both the necessity of a proper diagnostic pattern (test swabs) and the chance to manage asymptomatic patients. For this reason, every patient should be treated as infected to avoid any risk. To date, it is not proven that a patient that recovered from a previous infection to COVID-19 developed a complete and lasting immunity to the disease. In fact, reactivation of the disease [34] or even reinfection have been reported [35]. Our suggested preventive measures should be followed until herd immunity is achieved and the following patient categories are properly identified:

(A) COVID-19 Symptomatic patient;
(B) Asymptomatic positive patient;
(C) Recovered patient that was previously symptomatic;
(D) Recovered patient that was previously asymptomatic;
(E) Negative Patient:
  - Very high systemic risk **
  - High systemic risk *
  - No systemic risk [36]

** Transplant patients, cancer patients, people with severe respiratory conditions including cystic fibrosis, severe asthma and severe chronic obstructive pulmonary, people with rare diseases and inborn errors, people on immunosuppression therapies, women who are pregnant with significant heart disease (congenital or acquired) [36].

* People aged 70 or older, people under 70 with an underlying health condition (chronic respiratory diseases, chronic heart disease, chronic kidney disease, chronic liver disease, chronic neurological conditions, diabetes, problems with spleen, a weakened immune system due to conditions such as HIV and AIDS or medicines, a body mass index (BMI) of 40 or above), pregnant women [36].

#### 1.2.3. Experience of a Single Center

To support clinicians in dental management during the COVID-19 epidemic, we want to share our two months of experience based on a workflow centered on the following key points:

(1) Assessment of the patient risk level based on a multiparameter analysis related to dental chief complaint, history of COVID-19 exposure, and systemic conditions.
(2) Promotion of a two-phase dental procedure management: (a) remote contact via telephone and/or web for preliminary risk level evaluation and telediagnosis, and (b) face-to-face treatment.
(3) Use of the updated preventive measures adopted in a COVID Hub Hospital for dental interventions with patient’s centered spaces and times of health care.

## 2. Materials and Methods

The following preventive strategies were adopted in an Italian University Hospital Dental School during COVID-19 era (updated to 23 April 2020). These measures were approved by the University Hospital of Messina, which currently hosts a dedicated COVID-19 hub for the management of an area with about 627,000 citizens, and focus on the management of operators, patients, environments, as well as instruments.

The Dental Unit is provided with 20 dental chairs on two different floors. Three distinct operative areas were identified, one on the first floor of the clinic, the remaining two on the second floor. Each operative area is provided with 4 dental chairs. In case of multiple dental chairs in the same room, only one was rendered active at a time.

### 2.1. Concept of Urgent and Postponable Dental Procedures during Epidemic

It is well known that the majority of dental restorative, prosthetic and periodontal procedures are considered elective because they are planned and scheduled in advance. In order to avoid a supplemental infectious risk, they must be possibly postponed in all countries where the COVID-19 epidemic is present until the acute phase ends.

On the other hand, some dental pathologies require urgent treatment, even during an epidemic.

Preliminarily, it is necessary to clarify the concept of urgent and postponable procedures. We assumed the ADA report [37] regarding “urgent” treatments that can be practiced during COVID-19 epidemic. They can be divided into three categories:

**Category** **1.** *Dental emergencies that are potentially life threatening that require immediate treatment (within 1 h).*

*(1A)*  *Uncontrolled bleeding.*
*(1B)*  *Diffused soft tissue infection with intra-oral or extra-oral swelling that potentially compromise patient’s airway.*

**Category** **2.** *Urgent dental care (within the 24 h), conditions that require immediate attention to relieve severe pain and/or risk of infection and to alleviate the burden in hospital emergency departments.*

*(2A)*  *Severe dental pain from pulpal inflammation.*
*(2B)*  *Pericoronitis or third-molar pain.*
*(2C)*  *Surgical post-operative osteitis.*
*(2D)*  *Abscess, or localized bacterial infection.*
*(2E)*  *Tooth fracture resulting in pain or causing soft tissue trauma.*
*(2F)*  *Dental trauma with avulsion/luxation.*
*(2G)*  *Dental treatment required prior to critical medical procedures.*
*(2H)*  *Final crown/bridge cementation if the temporary restoration is lost, broken or causing gum irritation.*
*(2I)*  *Biopsy of abnormal tissue.*

**Category** **3.** *Undeferrable treatments (more than 24 h).*

*(3A)*  *Extensive dental caries or defective restorations causing pain.*
*(3B)*  *Suture removal.*
*(3C)*  *Denture adjustment on radiation/oncology patients.*
*(3D)*  *Denture adjustments or repairs when function impeded.*
*(3E)*  *Replacing temporary filling on endo access openings in patients experiencing pain.*
*(3F)*  *Snipping or adjustment of an orthodontic wire or appliances piercing or ulcerating the oral mucosa.*

**Category** **4.** *Non-urgent treatments.*

*(4A)*  *Initial or periodic oral examinations and recall visits, including routine radiographs.*
*(4B)*  *Routine dental cleaning and preventive therapies.*
*(4C)*  *Orthodontic procedures.*
*(4D)*  *Extraction of asymptomatic teeth.*
*(4E)*  *Restorative dentistry including of asymptomatic carious lesions’ treatment.*

Our advice is pointed toward the non-urgent treatments, assuming that even non-urgent procedures will be eventually reintegrated in the common practice, preferably once a better understanding of the COVID-19 immune response is achieved. While ADA suggested in its Interim Guidance [38] to delay the treatment of non-urgent dental procedures indefinitely during the COVID-19 acute phase, we do not believe this behavior will fit the post-acute phase of the epidemic. Clinicians should be ready to manage non-urgent dental care to avoid a worsening in the clinical conditions that may lead to urgent or non-treatable scenarios.

Patients appointments should be limited in number, aiming to group as many procedures as possible in the single access. This behavior would lead to a more cost-effective practice, mostly due to an optimized use of the protective gear that could otherwise be expensive.

For instance, we suggest to complete endodontic treatments in the fewest number of appointments, while restorative and surgical procedures should be performed per quadrant. Use of resorbable sutures is recommended. Aerosol-generating procedures, like air-flows or tooth preparation for dental prosthesis, represent the critical moment in the dental practice in the COVID-19 era and should be managed carefully and performed last.

### 2.2. Operators Management

Four units were involved in each patient dental management:

(a) One administrative staff member;
(b) One nursing staff member outside the operative area;
(c) One nursing staff member inside the operative area;
(d) One clinician.

In the private practice, the nursing staff outside the operative area could simultaneously carry out the administrative role, such as secretarial work, reception duties, and payments.

All operators followed the same prevention rules. Respecting good personal hygiene rules was an essential requirement. A shower before going to work and after activities will certainly be a recommended practice [39].

Our advice is to shave facial hair, to keep the fingernails short, and avoid the use of any accessories such as watches, rings, bracelets, etc. It is also advised to wash hands with alcohol-based hand sanitizer for at least 20 s before and after each treatment, and limit contacts with surfaces, computers, drawers etc., as much as possible. Moreover, clinicians should avoid touching their faces, including eyes, nose and mouth. Sterile preparation criteria should be applied on every step of the clinical practice, including the operator dressing–undressing routine. The clothing must include: shoe covers, disposable caps, disposable waterproof gowns, disposable gloves, protective glasses and visors, and protective masks.

Staff members were dressed as specified in Table 1.

As per the respiratory protection masks, a certified high filtration percentage (>94%) is recommended, such as Europe “filtering facepiece 2” (FFP2), U.S. “NIOSH N95”, China “KN95”, Australian/New Zealand “P2”, Korea “1st class”, or Japan “DS” [40]. We suggest the use of very high filtration (>99%) facial devices, like FFP3 masks. A brief comparison between FFP protective devices is shown in Table 2 [41].

Due to the fact that the patient may still produce droplets by coughing or sneezing, and that airborne transmission has not been ruled out as a spreading mechanism, a safe and cautious approach is then strongly advised. We have adopted protective glasses and visors and FFP3 masks even when performing non-aerosol-generating procedures. Protective glasses and visors must be disinfected with 70% ethyl alcohol before and after every treatment. Alternative protective head gear designs have been proposed; among them, protective masks based on models used for snorkeling and diving, completely cleanable and equipped with interchangeable disposable P3 filters, have been developed by the Engineering Department of the University of Messina, and look promising. A critical issue of this adapted devices could be represented by the fact that eyeglasses, magnifying systems, and lighting systems are rendered impossible to wear.

### 2.3. Patient Management

An updated Italian review of the literature regarding dental care in the COVID era identified four phases in the patient management: patient triage, patient admission into the practice, dental treatment, and patient discharge [42]. To highlight these four key moments, we opted for a two-step patient management: remote (patient triage) and face-to-face (patient admission into the practice, dental treatment, patient discharge).

**Figure 1:**
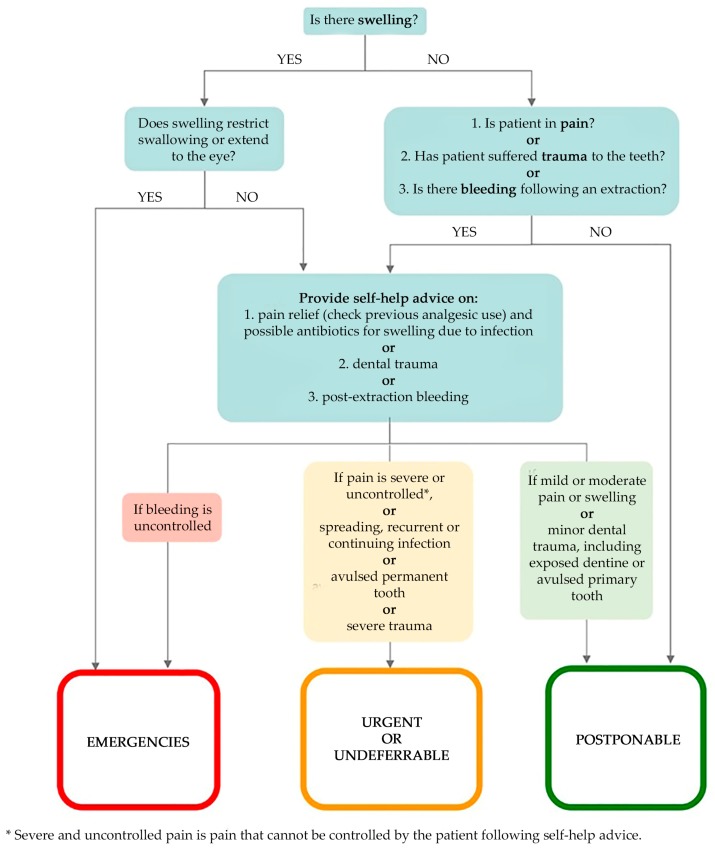
Flow chart of “urgency of treatment” category assignation, a modified version of the one proposed by SDCEP [43].

In the first step, every patient was managed remotely (e.g., phone, texts, website) adopting a dental triage that consists of an interview able to identify three parameters:

- Chief complaint in order to identify emergencies, urgent, undeferrable and/or postponable dental procedures (Figure 1):
  1. “Emergencies” (Category 1 ADA): within 1 h, managed through E.R;
  2. “Urgent” (Category 2 ADA): within 24 h;
  3. “Undeferrable” (Category 3 ADA): possibly more than 24 h;
  4. “Postponable” (Category 4 ADA): to be treated remotely.
- COVID-19 personal history obtained through the following questionnaire:
  (a) Are you or were you infected with COVID-19?
  (b) Have you had a fever, cough, cold, breathing difficulties, muscle pain or headache in the last 28 days?
  (c) Have you had contacts with individuals who have had these symptoms in the last 28 days?
  (d) Have you been in contact with infected individuals in the last 28 days?
  (e) Did you undergo a swab test that returned a positive result for COVID-19?
  (f) Have you been in a previously quarantined area?
  (g) Have you had contact with individuals coming from quarantined areas?
- Systemic risk category, as mentioned above:
  (A) COVID-19 Symptomatic patient;
  (B) Asymptomatic positive patient;
  (C) Recovered patient that was previously symptomatic;
  (D) Recovered patient that was previously asymptomatic;
  (E) Negative Patient:
    - Very high systemic risk
    - High systemic risk
    - No systemic risk [36]

The interview may be supplemented with additional data, such as personal medical records or clinical pictures that are transmitted to the clinicians via electronic means, such as e-mail, instant messaging platforms, etc. Once the interview has been completed, the clinician must choose the appropriate physical and/or temporal “patient separation protocol” [36].

In case of postponable procedures, remote treatment should focus on two key concepts: advice and self-help. Patient should be informed of the current preventive measure and separation protocols, and clinicians must provide advice and self-help, which might involve the prescription of antimicrobials and analgesics [43]. In case of a COVID-19 positive symptomatic patient requiring drug prescription, eventual antibiotic and analgesic therapy should be managed together with the clinician in charge of the patient (e.g., family doctor, oncologist, etc.).

Appointments were scheduled in order to admit a single patient per time inside the clinic areas, inviting patients to avoid the presence of unnecessary accompanying persons and to come wearing disposable gloves and mask.

Clinicians had to manage only one patient at a time, avoiding contact with other patients and granting a sufficient spawn of time for the work environment sanitation. Our advice is to identify a defined and specific pathway that patients from each risk category must follow whenever they transit inside the clinic rooms, from admission to discharge. Every movement has to be followed by the nurse for safety means.

In the second step, as soon as the patient arrived, they were admitted to a “standby area”. Social distancing measures were applied. A disposable mask was provided when not already worn. Nurse then registered body temperature without coming into contact with them, using infrared thermometers. The interview was then repeated and, in case of discrepancies with the info previously acquired, patient was eventually rescheduled to grant appropriate separation and prevention protocols, and informing the local COVID-hub. If the answers matched the ones previously registered, they were then invited to deposit their personal effects into a plastic bag that was sealed and stored in the standby area, and were admitted to the waiting room. Before entering the operating area, they were provided with an alcoholic hand sanitizer, disposable gloves, shoe covers, and a cap. Once accessed the operating area and before the exploration of the oral cavity, the patient had to remove the disposable mask, and was invited to rinse for 30 s with a 1% solution of hydrogen peroxide (a part of hydrogen peroxide 10 volumes/3% and two parts of water) or with 1% povidone iodine. Povidone-iodine may inactivate COVID-19, thus reducing its infectivity [44], and a protocol based on povidone-iodine nasal spray and mouthwash has already been proposed during the actual epidemic to reduce cross infection and protect healthcare workers [45]. After that, another mouth rinse with 0.2/0.3% chlorhexidine for 1 min was performed to reduce the bacterial load in the aerosol [46].

Once treatment was over, patient was discharged. During this phase, they were ordered to remove the previously provided disposable devices, put on another disposable mask, clean again their hands using an alcoholic hand sanitizer, and put on gloves. They were escorted to the discharge area, whereas eventual prescriptions and written advices were given. The patient was accompanied back to the standby area to retrieve their personal effects, remove the shoe covers, and exit the clinic. Every disposable device worn by the patient was considered as infected and had to be disposed accordingly.

Our advice to private dental practitioners is to refer the most challenging clinical scenarios to the Public Health Service. This refers to patient either with active fever (>37.2 °C), currently infected, and/or belonging to the very high risk category.

**Figure 2:**
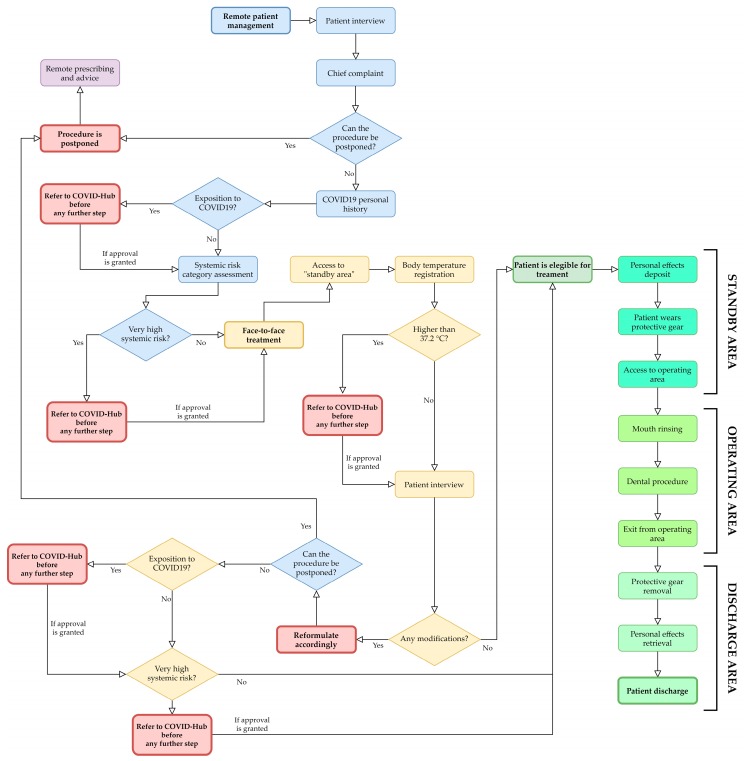
Proposed flow-chart for patient management.

A flow chart for the proposed patient management is shown in Figure 2.

### 2.4. Environments Management

The following rules result from our experience in the management of the clinical areas.

During the treatment, the work environment must be kept closed, and we suggest to implement in operating rooms ventilation and air filtration systems suitable for the health activity.

The access to the operating area must be restricted as much as possible, even to other staff members, if not strictly necessary. Moreover, no more than one patient should be treated in the same working space (e.g., rooms with multiple dental chairs), unless the working units are properly divided from one another. Environmental management should not be underestimated. Every surface in the waiting room must be considered at risk [33]; for this reason, it must be sanitized and properly ventilated before and after each appointment. All handles, seats and furniture should be cleaned. Everything that can come into contact with the patient (buttons, counters, chairs) has to be disinfected. Toilets must be sanitized before the entry of each patient and after his exit from the environment. They should be equipped with soap and alcoholic gel solutions for hand disinfection as well as disposable wipes. Operating areas and all surfaces must be disinfected before and after each treatment and be adequately ventilated.

Our suggestion is to choose rapid acting, broad spectrum disinfectants (Table 3), following manufacturer’s instructions whenever using them. In accordance with the provisions of the Hygiene and Preventive Medicine Unit of our University Hospital, surface cleaning was performed with 70% ethyl alcohol, followed by sanitation with potassium peroxymonosulphate solutions, alternating sodium hypochlorite 2.5% and 55% hydroalcoholic solution with quaternary ammonium propionate. Every product was applied using disposable tissues.

### 2.5. Instruments Management

The following measures were adopted during the last 6 weeks. Surgical field sterile preparation is strongly recommended for any dental procedure, even non-surgical ones. Whenever possible, every dental procedure must limit the amount of aerosol produced to a minimum, avoiding the use of the air-water syringe or scale. It is essential to always use a double high-speed aspiration and anti-reflux handpieces to limit the risk of cross-infections. Any single-use instrument must be properly disposed. Intraoral X-ray exams should be limited due to the stimulation of saliva production, preferring extra-oral dental exams such as panoramic radiography or cone beam CT [46]. Dental impression and models should be sanitized with alcohol-based cleaners. Digital impressions are highly suggested. Dental chair, handpieces, lamp and suction system hoses are managed as specified in Table 4.

### 2.6. COVID-19 Prevention in Home Environments

As previously mentioned, saliva is an important coronavirus vehicle, either by inhalation, ingestion, direct mucous contact with droplets, and by mucous contact with residual droplets on hands, objects or surfaces infected in the previous 2–9 days [47].

Preventive measures should address home environments firstly, and the clinician may offer useful suggestions on the behaviors to be implemented.

The advices leaflet given to the patients to the patient discharged from our unit are shown in Appendix A.

## 3. Results

The unit was able to manage up to 20 patients remotely and 12 patients face-to-face using the proposed workflow per working day, from 8 A.M to 2 P.M. Additionally, a small cluster of high risk and very high risk patients were admitted to the dental unit during the two-month time span, to perform either clinical examinations or schedule future treatments.

Remote management enabled clinicians diagnose and eventually treat a wide spectrum of clinical scenario, ranging from oral infections to more severe conditions, like a case of necrotizing fasciitis that resulted in the patient’s transferal to the Reanimation Unit after the diagnosis. No COVID-19 positive patients were treated. While following the proposed workflow, no COVID-19 new cases were detected among patients and staff.

## 4. Discussion

To date, no universal guideline regarding dental management during the COVID-19 epidemic is available. While some National Health Institutions, like NHS, started providing guidance and advices for the management of clinical urgencies during the pandemic, the lack of clear standards severely affected the dental care services [48]. The assessment of the patient risk level through analysis of dental chief complaint, history of COVID-19 exposure, and systemic conditions is the first necessary step to safely manage patients during the epidemic.

Remote management should always precede the face-to-face treatments in order to avoid useless direct contacts that might only increase the risk of infection for both patients and clinicians.

The proposed workflow granted an appropriate management of patients, dental staff, environments, and instruments. Being effective and straightforward, it ensured the possibility to treat up to 12 patients per working day in a dental unit provided with three different areas for dental procedures.

## 5. Conclusions

This paper describes the two months experience of an Italian School of Dentistry into the development of infection prevention strategies during the COVID-19 epidemic (updated to 23-04-2020). The resulting workflow was realized with consideration of the most recent literature regarding COVID-19 in dental practice, with a strong influence from other Italian experiences [42]. Our aim is to share our choices regarding the management of operators, patients, environments, and instruments in order to provide a useful tool when entering the post-acute phase of the epidemic.

Understanding the role of dental environments in COVID-19 transmission may have a positive impact in the prevention of infection. Workplaces as well as home environments must be considered at risk. In order to decrease the possibility of contagion, it will be necessary to keep sanitized the work environments and sterile the instruments, taking the right precautions in the management of patients and operators. Even paying attention to home devices for daily oral hygiene might have a positive impact on maintaining individual health and lower the chances of spreading the infection. Furthermore, it would be advisable to increase patient awareness, clarifying the spreading characteristics of the virus and how it can be possible to fight and stop the propagation of COVID-19 in a time when infection could be fatal.

## Figures and Tables

**Table 1:** Personal protective equipment under different scenarios.

DPI	Outside the Operative Area	Non-aerosol Generating Procedures	Aerosol Generating Procedures
**Surgical mask**	√		
**FFP2/FFP3 mask**		√	√
**Face shield**			√
**Protective glasses**		√	√
**Gloves**	√	√	√
**Cap**	√	√	√
**Protective waterproof clothing**			√
**Shoe cover**		√	√

**Table 2:** Aerosol filtration percentage and internal leak rate for FFP masks.

Types of Masks	Specifics
FFP1	Aerosol filtration percentage: 80% minimum Internal leak rate: Maximum 22%
FFP2 (equivalent to N95)	Aerosol filtration percentage: Not less than 94% Internal leak rate: Maximum 8%
FFP3	Aerosol filtration percentage: Not less than 99% for EN 149-FFP3. And 99.95% for EN 143-P3 Internal leak rate: Maximum 2%

**Table 3:** Broad spectrum chemicals for environment disinfection.

Disinfectant	Required Exposure Time
70% ethyl alcohol	5 min
Potassium peroxymonosulphate solution (1/100 dilution)	5 min
2.5% sodium hypochlorite	5 min
55% hydroalcoholic solution with quaternary ammonium propionate	5 min

**Table 4:** Instruments management in aerosol/non aerosol-generating procedures.

Preventive Measures/Tools	Non Aerosol-Generating Procedures	Aerosol-Generating Procedures
1. Sterility rules for any autoclavable instrument	YES	YES
2. Protect dental unit, lamp, handles with disposable films that will be properly disposed at the end of the session	YES	YES
3. Protect handpieces, hoses of handpieces, hoses of suction system, handles, trays and shelves with disposable films	NO	YES
4. Necessary tools only on the shelves	YES	YES
5. Rubber dam	NO	YES

## Appendix A

Dear patient,

The following suggestions and advices aim to reduce the risk of COVID-19 infection that you may incur when sharing your home environment with other people.

(A) Hand-washing

Coronavirus is also transmitted through the hands [47]. This concept is not often kept in mind when dedicating to daily oral self-care. As already recommended also by WHO [49], it would be useful to preliminarily wash hands properly.

Social hand-washing is useful in these situations; we list the main steps [50]:

Wet and lather both hands and wrists, thoroughly clean all parts, including interdigital and nail spaces for 60 s, rinse thoroughly with running water, dry properly with a disposable towel, and use the disposable towel to close the tap.

(B) Replace toothbrush and Toothpaste

To avoid contagion with previously infected saliva, it would be advisable to replace the toothbrush, toothpaste and floss with new ones. Sharing even one of the aforementioned medical devices for home oral hygiene among family members could expose every component to the risk of contagion. Each member of the family should have their own toothbrush, preferably of a different color from the others, as well as their own toothpaste and dental floss. Similarly, the handpiece of the electric toothbrush or Waterpik should not be shared.

(C) Rinse toothbrush

After brushing teeth correctly, carefully remove all the toothpaste residues with hot water. These residues can retain microorganisms after brushing.

(D) Pay attention to glasses or containers for toothbrushes

In families, people often share the same environment for daily personal hygiene. Mistakenly, in a home environment, we used to put toothbrushes or the heads of the electric toothbrushes of all family members inside the same glass or tray. This is an incorrect practice, since it would expose others to the risk of infection. Any infected saliva on the handle or on the bristles could come into contact with the other toothbrushes, infecting them. For this reason, it is suggested to prepare a dedicated glass for each member of the family, placed far enough from the others. This will ensure that any contact will be avoided. It would also be advisable to avoid storing toothbrushes inside closed containers or drawers; in these environments, it is difficult to maintain infection control. It is therefore recommended to keep toothbrush inside clean glasses without using caps, especially if previously used.

(E) Getting sick requires starting over

Replacing everything and properly cleaning surfaces and medical devices would be the optimal solution in case of sickness.
